# A Python package based on robust statistical analysis for serial crystallography data processing

**DOI:** 10.1107/S2059798323005855

**Published:** 2023-08-16

**Authors:** Marjan Hadian-Jazi, Alireza Sadri

**Affiliations:** a The Walter and Eliza Hall Institute of Medical Research, Parkville, Melbourne, Victoria 3052, Australia; b Department of Medical Biology, The University of Melbourne, Parkville, Melbourne, Victoria 3052, Australia; c School of Physics and Astronomy, Monash University, Clayton, Victoria 3800, Australia; STFC Rutherford Appleton Laboratory, United Kingdom

**Keywords:** *RGFlib*, robust statistics, serial crystallography, robust peak-finding, robust bad pixel mask making

## Abstract

The term robustness in statistics refers to methods that are generally insensitive to deviations from model assumptions. In other words, robust methods are able to preserve their accuracy even when the data do not perfectly fit the statistical models. Robust statistical analyses are particularly effective when analysing mixtures of probability distributions. Therefore, these methods enable the discretization of X-ray serial crystallography data into two probability distributions: a group comprising true data points (for example the background intensities) and another group comprising outliers (for example Bragg peaks or bad pixels on an X-ray detector). These characteristics of robust statistical analysis are beneficial for the ever-increasing volume of serial crystallography (SX) data sets produced at synchrotron and X-ray free-electron laser (XFEL) sources. The key advantage of the use of robust statistics for some applications in SX data analysis is that it requires minimal parameter tuning because of its insensitivity to the input parameters. In this paper, a software package called *Robust Gaussian Fitting library* (*RGFlib*) is introduced that is based on the concept of robust statistics. Two methods are presented based on the concept of robust statistics and *RGFlib* for two SX data-analysis tasks: (i) a robust peak-finding algorithm and (ii) an automated robust method to detect bad pixels on X-ray pixel detectors.

## Abstract 2

This article introduces *RGFlib*, a Python package for robust statistical analysis. The package is a useful tool for a variety of tasks in X-ray crystallography data analysis, such as peak-finding, bad pixel mask making and other outlier-detection tasks.

## 1. Introduction

In X-ray crystallography, statistics are used, among other things, to describe data structures such as Bragg peaks in a data set. Two popular examples of statistics that are used very often are the standard deviation and the data average. These statistics are called non-robust statistics. Non-robust statistics are useful when the data set contains only a single data structure and does not contain outliers. In such a case, maximum-likelihood estimation can be used for data processing and to fit a model to data (Meer, 2004 ▸). In such applications and in order to use non-robust statistics, outlier-rejection methods must be used, since non-robust statistics are sensitive to outliers. However, if the data are contaminated with outliers, non-robust statistics cannot be used directly on the entire data set to provide a model for data. In such cases, another type of statistics called robust statistics can be used (Rousseeuw & Leroy, 1987 ▸).

In the statistics literature, a set of data points from noisy measurements is called a data set. The problem of estimating a model for a data set (with noisy data points) is called geometric model fitting. Depending on the dimension of the data points, the geometric model can be a constant (*a*) for 1D data, a line (*a* + *bx*) for 2D data, a plane (*a* + *bx* + *cy*) for 3D data and so on. In these cases *a*, *b*, *c*, … are called model parameters and the target estimation model is called the structural model or data structure. Robust statistics have proven to perform reliably in the presence of outliers (Huber, 2011 ▸). One of their main applications is to detect multiple structures in data. Robust statistics can also perform well when the noise densities are modelled with skewed densities such as the Poisson probability density function. The median is a popular example of a robust statistic, even though it is a biased estimator with low efficiency.

In this paper, we introduce a software library package based on modern robust statistical and clustering methods named *Robust Gaussian Fitting library* (*RGFlib*). The functions implemented in the library can help in the efficient analysis of different data types including X-ray diffraction patterns. *RGFlib* can also be used by existing data-analysis software suites, as implemented in the popular Python and C programming languages. Furthermore, we will discuss how robust statistical methods can help to improve the data quality in the analysis of X-ray serial crystallography (SX) diffraction patterns. The aim of this paper is to demonstrate the use of robust statistics in serial crystallography and to describe the source code of the *RGFlib* Python package and how to use its functions for data analysis.

## 2. Methods

**Figure 1:**
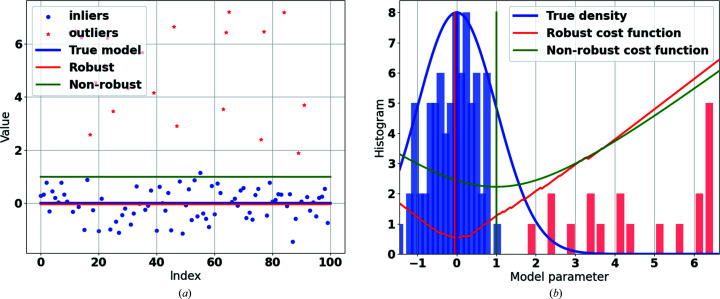
An example of fitting a geometric model to a data set. (*a*) The true data points (blue markers) are samples from a Gaussian distribution and their density is shown in blue in (*b*). The red markers are outliers and are samples from a uniform distribution in the range [1.5, 6.5]. The blue horizontal line on the left shows the true model (line). The green line shows the result of using non-robust statistics and the red line shows the result of using robust statistics. (*b*) The blue bars are the density of true data points and the red points are the density of outliers. The red curve shows the cost function used for the robust method and the green curve shows the cost function for the non-robust method. As can be seen, the cost function of the robust method has its optimum value where the density is close to maximum.

In order to clarify the difference between robust and non-robust statistics, an example of the application of robust statistics for data analysis is provided as follows. Assume a one-dimensional (1D) data set with a single structure (a cluster of data values) with Gaussian noise and outliers as shown in Fig. 1 ▸(*a*), where the outliers are shown in red. In this case the data points are noisy measurements from the true value 0 (the model parameter is *a* = 0). To generate this data set, we sampled from a Gaussian probability density function shown as a blue curve in Fig. 1 ▸(*b*) and added outliers. The histogram of true data points is also shown with blue bars and that of true outliers with red bars in Fig. 1 ▸(*b*). Here, the aim is to fit a model, in this case a single parameter (the mean of the Gaussian distribution), to data, find the scale (standard deviation) of the noise and label the outliers. Total least squares (TLS) is a popular non-robust method used for analysing data with rare outliers. It is widely used in this type of analysis. The mean-shift algorithm, on the other hand, is a similar but robust method for this type of outlier-contaminated data analysis (Fukunaga & Hostetler, 1975 ▸). The cost function of TLS is defined as the sum of the squared algebraic distance of every data point to the model (Meer, 2004 ▸). The single model parameter can be calculated by minimizing the cost function. Robust methods and their cost functions will be introduced later. In this example, the cost functions for non-robust and robust methods are shown as green and red curves, respectively, in the parameter space in Fig. 1 ▸(*b*). The model parameters calculated using non-robust and robust methods are shown in Fig. 1 ▸(*b*) as green and red vertical lines, respectively. This figure shows how outliers shift the model and make the estimated model inaccurate when using the non-robust method. Therefore, a robust statistic that can perform reliably and accurately in the presence of outliers is required to find the model parameters.

## 3. *Robust Gaussian Fitting library* (*RGFlib*)

Many challenges in the crystallographic data-analysis pipeline can be traced back to outlier detection. An example of outlier detection is finding Bragg peaks in X-ray crystallographic diffraction patterns. We have developed a software library to perform outlier detection, called the *Robust Gaussian Fitting library* (*RGFlib*; Sadri & Hadian-Jazi, 2020 ▸). The following section provides an overview of the *RGFlib* software, including its functions and input parameters. The default values of the input parameters are based on the statistical and crystallographic literature, and will be explained for each function. *RGFlib* is a software package developed in the C and Python programming languages that includes robust statistical functions which will be explained in this paper. The library is useful in many stages of the crystallographic data-analysis pipeline such as Bragg peak detection, bad pixel detection, data calibration, data reduction, indexing and merging. Here, we provide two examples where *RGFlib* was used for outlier detection in serial crystallography: Bragg peak detection and bad pixel detection in X-ray diffraction patterns. In Bragg peak and bad pixel detection, proper modelling of the background intensities leads to accurate results.

*RGFlib* is designed to provide robust statistical functions for separating out outliers in a data set using geometric models. Using these functions requires very few assumptions about the data, which will be explained later in this section. This is one of the main advantages of robust statistical methods. In this section, we will introduce the functions that are implemented in *RGFlib*, their input and output and the assumptions that are needed for the implemented algorithms to perform efficiently.

### 3.1. Robust model fitting

**Figure 2:**
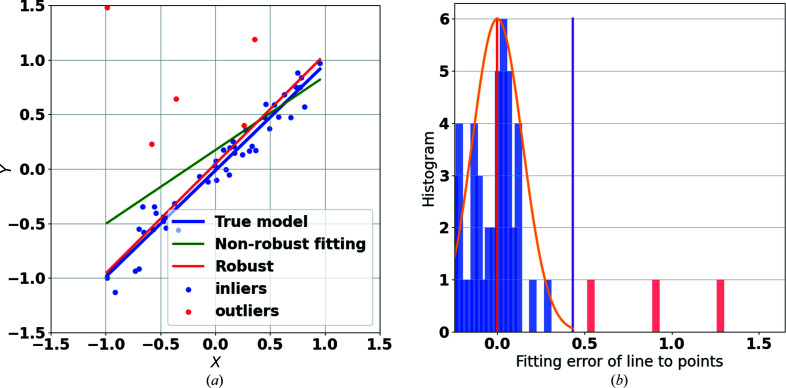
An example of geometric line fitting in 2D space. (*a*) The blue line indicates the true model that the data are sampled from, the green line shows the model fitted to the data set which is shifted away by outliers (red circles) from the true model and the red line shows the fitted model using *RGFlib* (with the fitLine function), which is closer to the true model (blue line) than the non-robust model (green line). (*b*) Histogram of data-point distances from the model; the orange curve shows the Gaussian model, the red line shows the average of the Gaussian density and the purple line shows the estimated threshold of true data points calculated by *RGFlib*. The blue bars are the density of the true data points and the red bars are the density of the outliers. The outliers can be seen on the right-hand side of the threshold.

An example of robust geometric model fitting is to fit a line to a set of 2D data points as shown in Fig. 2 ▸(*a*). The noise is modelled with a Gaussian probability density function in the presence of outliers that are uniformly independent and identically distributed.

The functions implemented in *RGFlib* include two main robust methods. The first is an optimization method called fast least *k*th-order statistics (FLkOS; Bab-Hadiashar & Hoseinnezhad, 2008 ▸) that optimizes data-structure estimation; an example of this is shown by the red line in Fig. 2 ▸(*a*). The second method is a noise-scale estimator called the modified selective statistical estimator (MSSE; Bab-Hadiashar & Suter, 1999 ▸) that calculates the standard deviation of the model.

The model fitted by the optimization method (FLkOS) and the scale calculated by the robust estimator (MSSE) are used to define a threshold to separate outliers from true data points. The fitted model is shown in the example by the red line and the threshold is shown by the purple line in Fig. 2 ▸(*b*).

#### 3.1.1. Minimum number of true data points for FLkOS

FLkOS is an optimization method that minimizes *L*_∞_ (the worst fitting error) of true data points (Bab-Hadiashar & Hoseinnezhad, 2008 ▸). The major challenge of *L*_∞_ minimization is that the size of the true data set is initially unknown. FLkOS can solve this problem if the user provides a rough estimate of the size of the target data structure. This estimate should be as large as possible, but must be smaller than the size of the target structure with certainty.

Assuming that a model is non-robustly fitted to a sample of true data points, sorting the data points by their fitting errors will position some of the true data points that are far from the model around the rough estimate of the structure size. This allows another sample to be choosen from the data set that is comprised of such data points. A model fitted to these data points non-robustly will naturally minimize their fitting error. This is repeated iteratively in FLkOS, which means that the final model will have the minimal fitting error for data points that are positioned around the structure size after sorting, and hence *L*_∞_ minimization is achieved.

In other words, given a set of *m*-dimensional data points *X* = {*x*(*j*)}, *j* = 1, …, *N*, where 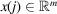 , and using a model with parameter θ, the goal is to segment out the outliers. As in Newton’s method (Huber, 2011 ▸), FLkOS minimizes the *L*_2_ norm cost function of a small subset of data, *i.e.* the parameters are optimized to minimize the fitting errors of a subset, rather than the whole data set. Among all possible subsets of *X* denoted by *e* here (*e* ∈ X), some have lower fitting errors. Assuming a subset *e*, the parameters of the model θ_*e*_ are calculated using the linear regression method by fitting the model to data points in *e*. The linear regression method minimizes 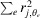 , where 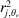 is the squared algebraic distance of the *j*th data point in *e* from the model with parameters θ_*e*_. Assuming θ_*e*_, the squared fitting errors, 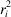 for the *i*th pixel, are calculated for all data points in *X*. The pixels in *X* are sorted according to their errors in ascending order.

The optimization algorithm chooses a new subset iteratively using a sorting step until it finds a subset that represents the target data structure. Data points are sorted according to their distance from the model of the previous iteration. Therefore, unlike Newton’s method, FLkOS is not sensitive to outliers as it uses guided sampling for each iteration. FLkOS is an optimization strategy that finds data points minimizing the cost function iteratively. This method takes the minimum number of true data points as input and finds the best fit regardless of the initialization.

To fit the model, it assumes that a percentile of data belong to the target data structure, while the aim of the method is to find out whether or not more data points are true data points. In other words, when sorted according to their distance to the estimate of the model, up to the *k*th farthest data point is assumed to belong to the model (for example the line in Fig. 2 ▸). The parameter *k* is the initial estimate of the number of true data points, which in *RGFlib* is called the ‘likely ratio’, and its default value is set to 0.5%. To obtain an interpretation of this parameter, for example in the Bragg peak-detection application, where the goal is to robustly estimate the average of background pixel intensities and separate them from Bragg peak pixels (outliers), this assumption means that within any reasonably large window in the diffraction-pattern image at least half of the pixels belong to the background.

#### 3.1.2. Gaussian cutoff threshold for MSSE

Assuming that our definition of the signal-to-noise ratio (SNR) can effectively represent the statistical separability of a Poisson density from outliers, the MSSE method can be used to calculate the variance of unknown true data and separate outliers. The following steps are used to segment out the outliers in a data set using the MSSE method. (i) The fitting errors ( 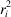 ) for all data points are calculated. This is performed by taking the squared difference between the actual value of the data point and the predicted value from the model. (ii) The fitting errors are sorted in ascending order (fitting errors are denoted 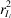 after sorting). (iii) After sorting, the MSSE method identifies all data points ordered after the 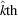 data point as outliers if 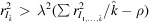 . Specifically, MSSE detects a data point as a true data point if its distance to the estimate from the model is no more than an input value (denoted λ) times the standard deviation of the Gaussian. This value is a global parameter in the algorithm, which is set between 2 and 4 in the statistics literature (Huber, 2011 ▸). This is based on the fact that more than 95% and 99% of a Gaussian density is within two and four times its scale (standard deviation), respectively. The points closest to the model are found by sorting them according to their distance to the estimation. This procedure is known as fitting a χ^2^ density to the squared errors (Bab-Hadiashar & Suter, 1999 ▸).

A traditional way of describing data points as outliers is by defining an SNR for each one of them along with an input threshold for the SNRs of true data points. There are many possible definitions, but we use that of statistical separability for SNR (Hadian-Jazi *et al.*, 2013 ▸). Therefore, given a data point with scalar value *x*_*p*_, a fitted Gaussian mean μ_*B*_ and a standard deviation σ_*B*_, the SNR is defined as SNR = (μ_*B*_ − *x*_*p*_)/μ_*B*_.

### 3.2. Functions for robust geometric model fitting

*RGFlib* uses the above definitions and methods to fit a model to true data points of a data set, which enables outlier detection. The library supports scalar value fitting and geometric model fitting (line and plane). In the case of value fitting it also supports modelling by a unimodal skewed density function. This is desirable for applications where the Gaussian is suspected to be modified by an exponential probability density function as a result of the presence of a Poisson process and system noise. In the following sections the functions of *RGFlib* are discussed.

#### 3.2.1. MSSE

As discussed earlier, the MSSE function is for the robust segmentation of true data points. The input of this function is a vector of fitting errors for all data points and a cutoff threshold for Gaussian density. For example, in Bragg peak detection (Hadian-Jazi *et al.*, 2021 ▸) the data points are the intensity values of pixels and the goal is to model the background intensities by fitting a plane. A fitting error, the distance of each pixel value from the plane (model), is calculated for each pixel. The pixels that are outliers to the plane will be singled out as Bragg peaks and the rest form a Gaussian distribution: the background intensities.

An example is provided to show how the software library can be used to segment out the outliers. Firstly, a noisy data set is generated. In this case, we assume that the variable dataset_1D includes the fitting errors of the data set, generated as follows: 
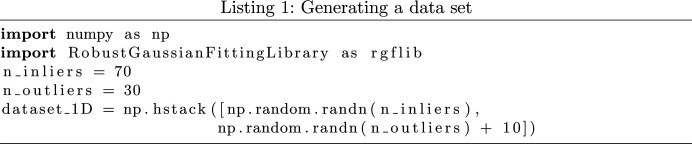


The numbers 70 and 30 are chosen arbitrarily in this example. The MSSE function from *RGFlib* can calculate the scale (standard deviation) of the noise in the data set: 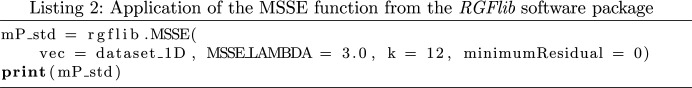


The first input of this function is the vector of the data set (which also includes outliers). The second input, MSSE_LAMBDA, is the threshold for fitting errors of true data points, as explained in Section 3.1.2, and is set to 3 by default (Sadri & Hadian-Jazi, 2020 ▸). *k* is the minimum number of true data points for a data structure, which is set to 12 by default (Hoseinnezhad *et al.*, 2010 ▸). A minimum for the absolute of the fitting errors is also available as an input, minimum­Residual, which is set to zero by default. If an input mask or a set of weights for each data point is available, one can use the function MSSEWeighted.

**Figure 3:**
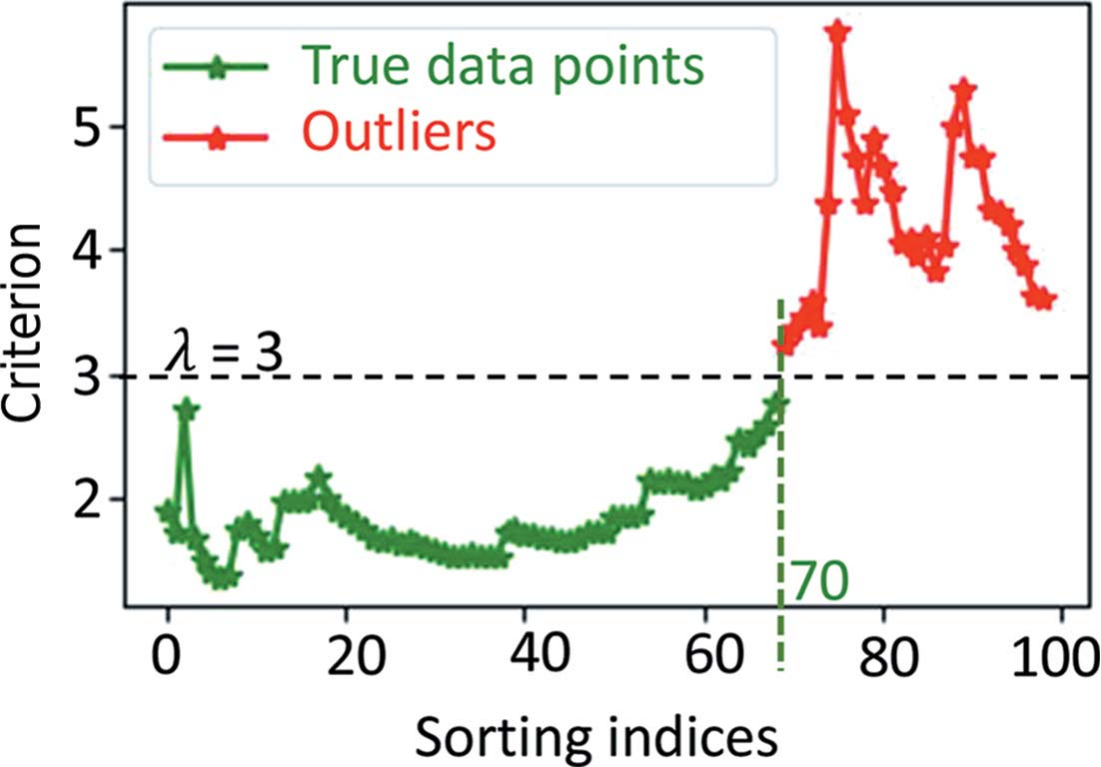
An example of the use of the MSSE function implemented in the *RGFlib* software package. The true data points are shown in green and the outliers are shown in red. In this example λ = 3.

Fig. 3 ▸ demonstrates an example of the use of the MSSE function implemented in the *RGFlib* software package that can be used for segmenting outliers. In this figure the outliers detected by MSSE are shown in red using the above Python code.

#### 3.2.2. fitValue

This function uses the FLkOS optimization method (Bab-Hadiashar & Hoseinnezhad, 2008 ▸) to fit a value to an input vector of data points. For example, this function can be used to fit a Poisson density to a region of an X-ray diffraction pattern to model the background intensity. The robust optimization method can be used to find the mean of the Poisson density without the impact of outliers. In the following an example of the usage of this function is provided. Here, the same data set from the MSSE example above is used (with 70% true data points and 30% outliers). The fitValue function from *RGFlib* can find the mean and standard deviation of data without the impact of outliers. An example of the application of fitValue is shown in Fig. 1 ▸. This figure was generated using the demo in the test module of *RGFlib* which uses the fitValue function as shown in the following code: 
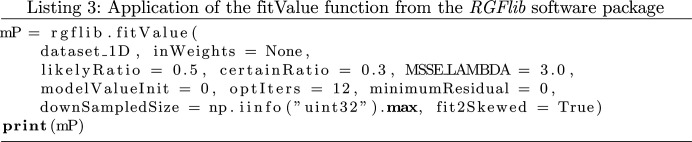


The input parameters for this function include likely­Ratio and certainRatio. likelyRatio is explained in Section 3.1.1 and by default is set to 0.5. certainRatio, which is set to 0.3 by default, gives that portion of the data set which is highly likely to be true data points. The sampling mechanism in *RGFlib* is initially affected by certainRatio. The fitValue function includes a feature (an input option which can be True or False) called fit2Skewed. If this is set to True, certainRatio will be reduced gradually until the number of optimization iterations reaches a predefined value (optIters). This enables the function to deal with skewed probability density functions (Sadri *et al.*, 2018 ▸).

**Figure 4:**
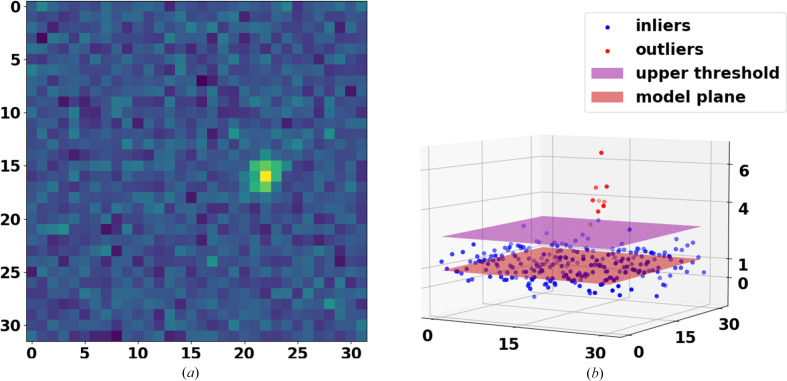
An example of plane fitting in 3D space. (*a*) Part of an X-ray diffraction pattern where a Bragg peak with high SNR is visible. (*b*) The pixel values are shown as data points in three dimensions. The red plane is the robust model fitted to true data points (blue markers) and those above the threshold (purple plane) are considered as outliers (red markers).

**Figure 5:**
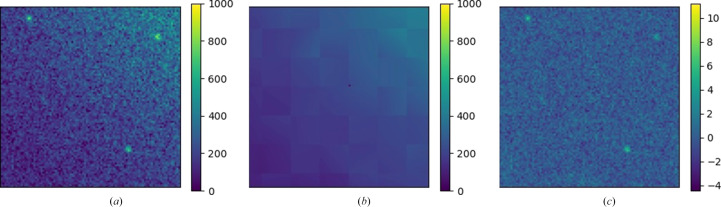
(*a*) A diffraction pattern with three Bragg peaks close to the water ring (the background intensity has a nonzero gradient). (*b*) The background intensity modelled by tilted planes using the *RGFlib* fitBackground function. (*c*) The diffraction pattern from which the background has been removed and every pixel has been normalized by the scale (standard deviation) of the noise. Bragg peaks are detectable after robust segmentation of the background.

modelValueInit enables initialization of the model parameters, which is disregarded by default. The output of fitValue is the robust mean and standard deviation of the target structure, modelled by a Gaussian density. A demo is provided in the test module of *RGFlib* to show the application of this optimization method (Sadri & Hadian-Jazi, 2020 ▸). Figs. 1, 2, 4 and 5 were generated using this demo and can be regenerated by running the Python code available in the test module on the *RGFlib* GitHub page (Sadri & Hadian-Jazi, 2020 ▸). Using downsampling, it is possible to speed up the method. In this case, downSampledSize can be used to define the size of the subset.

In order to increase the accuracy of the estimates, medianOfFits can be used. This method repeats the above process and calculates the median of the set of solution parameters as the result.

#### 3.2.3. fitLine

Given a 2D data set in Cartesian coordinates, this function fits a line robustly to the 2D data points. The function works similarly to fitValue but in 2D. The output includes three values: the slope and intercept of the line and the noise scale of the true data points. An example of the application of this function is shown in Fig. 2 ▸. The demo in the test module of *RGFlib* was used to generate this figure. The following code provides an example usage of the fitLine function from *RGFlib* in Python language: 
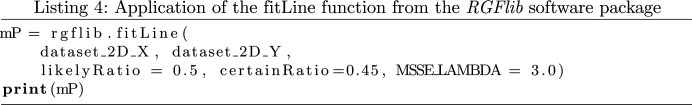


#### 3.2.4. fitPlane

Given a 3D data set in Cartesian coordinates, this function fits a plane robustly to the data points. This function works similarly to fitLine.

The output is composed of four parameters: three to define the plane (*ax* + *by* + *c*) and one to estimate the scale (standard deviation) of the true data-point noise. An example of the application of this function is shown in Fig. 4 ▸. Fig. 4 ▸(*a*) shows a simulation of part of an X-ray diffraction pattern image and Fig. 4 ▸(*b*) shows modelling the background of the image using fitPlane. This figure was generated using the demo in the test module of *RGFlib*.

The Python code for the example is as follows: 
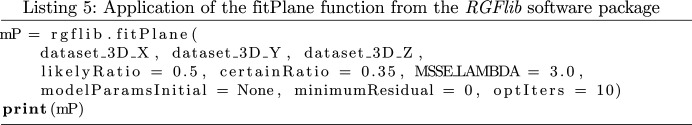


#### 3.2.5. fitBackground

This function takes an image as input and uses fitPlane to estimate the background intensity mean and standard deviation for each pixel in the image. The output can then easily be used to calculate the SNR for each pixel. An example is shown in Fig. 5 ▸. This figure was generated using the demo in the test module of *RGFlib* which uses the fitBackground function as shown in the code below. In this figure, the input image shows Bragg peaks close to the water ring. The image in this figure has been enlarged to show the peaks clearly; therefore, the ice ring is not visible. A horizontal plane cannot model the background properly, for example the mean or the median of the data. The SNR can be calculated more accurately if the plane is free to tilt according to the spatial gradient in the diffraction pattern. The fitBackground function from *RGFlib* can estimate the background of the image as a tilted plane. 
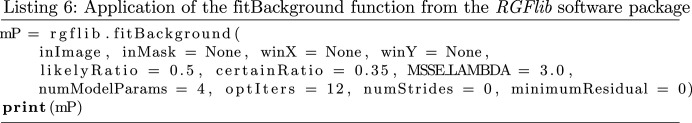


The above code shows an example of the usage of the function. The input of the function is an image. A mask for the image can be incorporated if required. To perform plane fitting to the background intensities, a window size can be set via winX and winY, which are by default set to the input image size. numModelParams can be set to 1 or 4. When it is set to 4, which is the default in the program, a tiltable plane (model) with three parameters will be estimated for the data. When it is set to 1 the data will be modelled with a horizontal plane. In machine learning, a sliding window is a window that slides across the data and performs a mathematical operation such as convolution. The distance that the window moves in each step is called the stride (Holbrook & Cook, 2022 ▸). Using sliding windows produces multiple maps for the background intensity values and noise scales. The average of these values provides an accurate estimate of the background model parameters. Therefore to increase the accuracy, numStrides can be used to set the number of strides of the window over the image. minimumResidual is the same as explained for the input of the MSSE function. The output of the function is two images with the same size as the input image. One is the model value at each pixel and the other is the scale of the noise for each pixel.

The optimizations mentioned above are implemented in the C programming language along with wrappers in Python to achieve a high processing speed. Moreover, most functions in *RGFlib* come in two forms: they can run in serial or parallel using the Python built-in multiprocessing package^1^. This can speed up the processes when used with cluster computers.

## 4. Applications of robust statistics in serial crystallography data analysis

In a serial crystallography experiment the samples are delivered via fixed-target holders (Frank *et al.*, 2014 ▸), a liquid jet (Berntsen *et al.*, 2019 ▸) or a tape such as Kapton or Mylar (Roessler *et al.*, 2013 ▸). The X-ray beam interacts with both the crystals and the jet and a diffraction pattern will be recorded on the detector (Chapman *et al.*, 2011 ▸). An example of a detector is the AGIPD-1M at the SPB/SFX beamline of the European XFEL (EuXFEL; Allahgholi *et al.*, 2015 ▸; Mancuso *et al.*, 2019 ▸). In a single SX experiment, hundreds of millions of diffraction-pattern images are produced; however, many of them do not include Bragg peaks and these patterns are called ‘non-hits’. Patterns that contain Bragg peaks are called ‘hits’ (Schlichting, 2015 ▸) and can be used to solve the structure of the crystal. The volume of data generated in an experiment can be significantly reduced if only the informative patterns (hits) are saved for further analysis. Therefore, it is very important to accurately separate hit and non-hit frames, a process called ‘hit-finding’. Hit-finding is achieved using a Bragg peak-detection method (called ‘peak-finding’ in the literature; Schlichting, 2015 ▸) and counting the number of detected peaks in each diffraction pattern. The *RGFlib* utility software package described here is a standalone Python package that can be used for data analysis. The following sections provide a brief overview of two use cases for the package in the field of SX. These use cases were previously published in Hadian-Jazi *et al.* (2021 ▸) and Sadri *et al.* (2022 ▸).

In the following section, we will discuss how we used robust statistics and *RGFlib* to improve the accuracy and speed of data reduction through effective hit-finding. We developed a peak-finding method called *Robust Peak Finder* (*RPF*; Hadian-Jazi *et al.*, 2017 ▸) based on the robust statistics implemented in *RGFlib*. We then evaluated the performance of *RPF* for data reduction in several experiments (Hadian-Jazi *et al.*, 2021 ▸).

We also applied *RGFlib* and modern robust statistical methods to another SX data-analysis step. We developed a method for making bad pixel masks called *Robust Mask Maker* (*RMM*) that detects bad pixels in X-ray detectors (Sadri *et al.*, 2022 ▸). Since hit-finding algorithms label a frame as a hit when the number of detected Bragg peaks therein is greater than a threshold, both peak-finding and making bad pixel masks are important steps in order to accurately identify hit frames. This makes the peak-finding algorithm a very important step in data reduction and also for the rest of the SX data-analysis pipeline. Identifying defective pixels in diffraction frames is a critical task, since peak-finding methods will detect the high intensity values of bad pixels in a pattern as Bragg peaks. Labelling bad pixels as peaks will lead to the storage of uninformative diffraction patterns as hits.

### 4.1. Robust statistics for peak-finding

An important goal in the serial crystallography data-analysis pipeline is to improve peak-finding and extract informative patterns so that we can avoid the storage of non-informative patterns through the hit-finding process. This will help to reduce the amount of data that is generated during each experiment in a facility such as EuXFEL. Detecting Bragg peaks accurately is also critical in order to perform further analysis such as indexing. There has been research to improve the accuracy and speed of peak-finding methods such as *CASS* (Foucar *et al.*, 2012 ▸), *Cheetah* (Barty *et al.*, 2014 ▸), *OnDA* (Mariani *et al.*, 2016 ▸) and *RPF* (Hadian-Jazi *et al.*, 2017 ▸). There has also been research in robust background modelling of diffraction patterns such as in the *DIALS* software package (Parkhurst *et al.*, 2016 ▸) and also in the *RPF* algorithm (Hadian-Jazi *et al.*, 2021 ▸).

We used *RPF* as an online monitoring method for data reduction (Hadian-Jazi *et al.*, 2021 ▸). One of the main advantages of *RPF* over existing methods such as *PF*8 (Barty *et al.*, 2014 ▸) is that the process of *RPF* can be parallelized. This is because modelling of the background is performed locally within a window around each candidate Bragg peak. *PF*8 (Barty *et al.*, 2014 ▸), for example, uses radial information for peak-finding and therefore requires geometry correction and access to information from all detector panels before the start of the process.

In the following, we provide a brief overview of how *RPF* uses robust statistics for the detection of Bragg peaks. *RPF* performs the detection of peaks from background in three main steps: (i) finding candidate peak pixels, (ii) modelling the background intensities robustly and estimating the SNR of the candidate pixel, and (iii) labelling candidate pixels and their neighbouring pixels as peaks if their calculated SNR is above a predefined threshold such as 6, which is the default value in *RPF*.

In the first step of *RPF*, a pixel is considered to be a candidate if its intensity value is a local maximum and is above a threshold, which is initially the median of the data within a local region. This threshold is updated as the background around the candidate Bragg peak is modelled more accurately. One hyper-parameter here is the size of the local window around a Bragg peak to model the background. The default size is 32 × 32 pixels for the AGIPD-1M detector. The fitted model is a plane and the SNR of the candidate pixel is calculated with respect to the data within this window.

In the last step of the algorithm, if the SNR of the candidate pixel is above a predefined threshold then all surrounding pixels above the background intensity will be labelled as part of the Bragg peak. The output of the algorithm is a peak list that includes the statistics of the detected Bragg peaks in each diffraction pattern.

**Figure 6:**
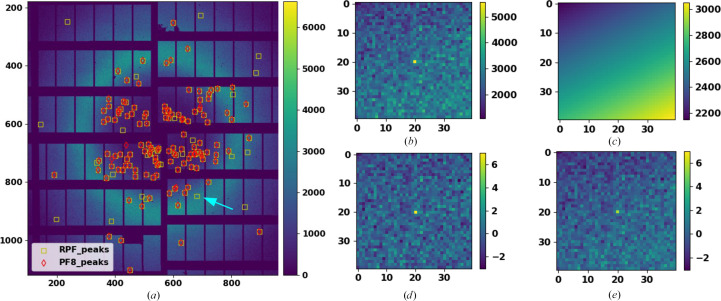
Analysis of a representative diffraction image from the EuXFEL data set. (*a*) A diffraction pattern chosen from the EuXFEL data set with peaks identified using *RPF* (yellow markers) and *PF*8 (red markers). (*b*) A Bragg peak and its local background that is detected with *RPF* and missed by *PF*8. (*c*) The estimated local background intensities with a tilted four-parameter plane using the *RPF* method. (*d*) Estimated SNR for a single Bragg peak isolated from the image in (*a*), as indicated by the arrow, using a robust method (*RPF*). (*e*) An estimated SNR for the same Bragg peak isolated in (*d*) but using the non-robust method (*PF*8). This figure has previously been published in Hadian-Jazi *et al.* (2021 ▸).

Fig. 6 ▸ provides an example of a diffraction pattern from the AGIPD-1M detector at the SPB/SFX beamline at the EuXFEL. Fig. 6 ▸(*a*) shows the Bragg peaks detected by both the *RPF* and *PF*8 methods. Fig. 6 ▸(*b*) shows an example of Bragg peaks that were detected with *RPF* and missed by *PF*8. Fig. 6 ▸(*c*) shows the estimated local background intensities using a tilted four-parameter plane, which was calculated using robust methods (*RPF*). Figs. 6 ▸(*d*) and 6 ▸(*e*) show the estimated SNR for each pixel surrounding the same Bragg peak using a robust and a non-robust method, respectively. These two figures show that the estimated SNR for the Bragg peak is 6.3 using the robust method and 5.8 using the non-robust method, which is below the SNR threshold of 6 (Hadian-Jazi *et al.*, 2021 ▸).

The main implementation of the *RPF* code is in C to achieve high speed. It inherits two functions from *RGFlib*: (i) MSSE to calculate the noise scale and (ii) the robust fitting of a tilted plane to pixel intensities in a given window of a diffraction pattern, as explained for fitPlane.

We tested the performance of *RPF* on many data sets, both real and simulated, and compared different crystallographic statistics such as CC*, SNR and *R*_split_, obtained using the *CrystFEL* package (White *et al.*, 2012 ▸), of the output with those obtained using established methods, especially using *PF*8 from the *Cheetah* package (Barty *et al.*, 2014 ▸). The results are presented in Hadian-Jazi *et al.* (2021 ▸). These results suggest that conventional methods might miss weaker Bragg peaks. If the sensitivity of non-robust methods is increased in order to detect weaker Bragg peaks, noise will also be detected as Bragg peaks. The results in Hadian-Jazi *et al.* (2021 ▸) show that *RPF* can achieve effective data reduction and can also provide accurate and reliable detection of Bragg peaks.

### 4.2. Robust statistics for bad pixel mask making

In this section, we will provide a brief overview of how robust statistics are used in another application in SX data analysis. In an SX experiment most of the diffraction patterns are non-hit and they do not include crystal structure information. However, in many cases the X-ray pixel detectors have bad pixels that generate false intensities that appear as Bragg peaks to the peak-finding algorithms. The uninformative pattern will then be assumed to be a hit by the hit-finding algorithm and will be stored for the next step of analysis (Sadri *et al.*, 2022 ▸). Bad pixels can also have negative effects on the performance of indexing methods and prevent them from finding crystals in hits. Some bad pixels of X-ray detectors can produce large intensity values. Some pixels may be permanently dead due to damage to the circuitry, and sometimes there are run-time artefacts such as shadows (Sadri *et al.*, 2022 ▸). It can happen that an entire panel randomly generates only noise that appears as meaningful signal. Such pixels show abnormal behaviour compared with healthy ones.

**Figure 7:**
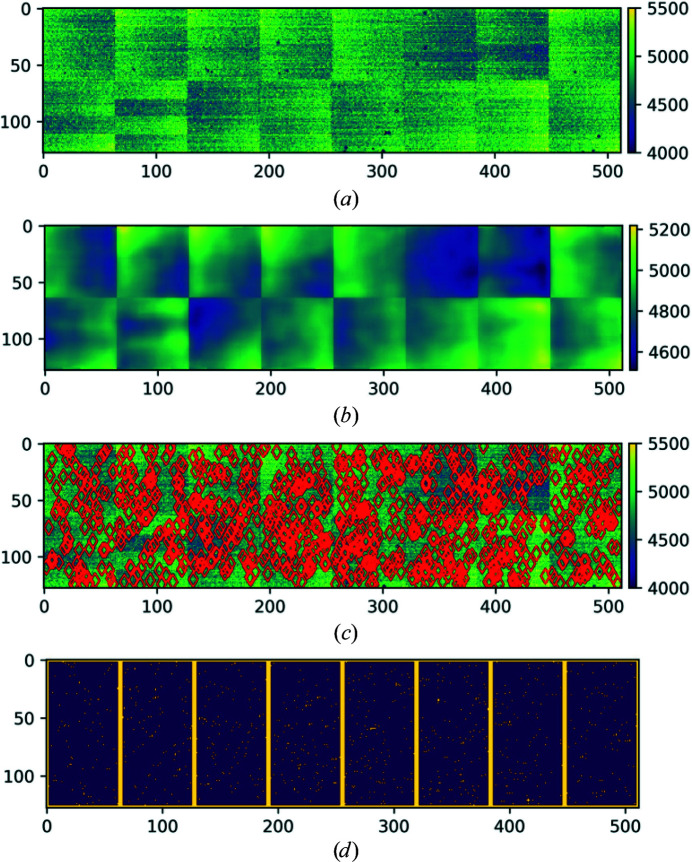
(*a*) The temporal average values of a module of an AGIPD-1M X-ray pixelated detector read in the absence of illumination. (*b*) The robust model values at the location of each pixel estimated to declare normal behaviour (estimated over pixels within 64 × 64 pixel windows). (*c*) The detected bad pixels are marked with red circles over the image from (*a*). (*d*) Detected bad pixels are shown in yellow. This figure has previously been published in Sadri *et al.* (2022 ▸).

In order to make a bad pixel mask for the X-ray detector, the data sets that are often collected for calibration and data-correction purposes are used. In such data sets, it is a reasonable assumption that all healthy pixels produce almost the same intensity values, *i.e.* these values can be modelled according to a geometric model with a Gaussian noise. Such data sets are collected when the detector is left in the dark or when a sample is used for imaging that generates a near-flat bright field, such as thin copper plates (Sadri *et al.*, 2022 ▸). An example of such a data set is shown in Fig. 7 ▸(*a*).

On such data sets, we used robust statistics to detect those pixels that behave abnormally. *RGFlib* can be used to model normally behaving pixels and calculate a statistical separability (SNR) for all pixels. Those pixels with an SNR above a predefined threshold (such as 6 in SX data analysis) are labelled bad pixels (abnormal). After the bad pixel mask has been made, the labelling can be used to exclude bad pixels from analysis, which will improve the performance of the peak-finding algorithm in the next step. This leads to a reduction of the stored data in the hit-finding step and also improves accuracy in the analysis. We implemented a pipeline that incorporates *RGFlib* to make bad pixel masks automatically called *Robust Mask Maker* (*RMM*; Sadri *et al.*, 2022 ▸).

The first step of *RMM* (Sadri *et al.*, 2022 ▸) is to extract statistical features for every pixel. These features describe the intensity values that pixels have under the dark field or the near-flat bright field. We called these features pixel abnormality maps. There are many different statistics for each pixel that have data features which can help in segmenting pixels into true data points and outliers. Features are obtained using non-robust statistics, allowing this to be biased by abnormal values that the pixel shows under the dark field or the near-flat bright field. An example of these features that can be used to make abnormality maps is the temporal average, which is supposed to be almost the same for all pixels. An example is shown in Fig. 7 ▸(*b*), which is used to produce a bad pixel mask for the AGIPD-1M X-ray detector.

In order to segment pixels into true data points and outliers, those that have similar features (true data points) can be modelled using a Gaussian distribution. As such, all generated features can be modelled robustly using functions from *RGFLib*. This enables the calculation of the SNR for each pixel with different statistical features to produce an abnormality map. Based on the abnormality map, those pixels with an SNR higher than a threshold are considered as outliers and are masked as bad pixels (Sadri *et al.*, 2022 ▸).

The output file of *RMM* contains an eight-bit value for every pixel. A value of 0 indicates a good pixel. Values above 0 indicate a specific problem for each pixel. *RMM* is implemented in Python and is publicly available for the generation of bad pixel masks (Sadri, 2021 ▸). One of the key benefits of *RMM* is that each module of the detector can be analysed in parallel, making the algorithm scalable on cluster computers. We have evaluated the performance of *RMM* for different data sets collected using AGIPD-1M, CSPAD-2.6M and PILATUS 6M detectors and showed that *RMM* improves data reduction and also the quality of SX data analysis (Sadri *et al.*, 2022 ▸). Figs. 7 ▸(*c*) and 7 ▸(*d*) show the implementation of *RMM* for detecting bad pixels of a module of the AGIPD-1M X-ray detector.

## 5. Summary

In this paper, we provide an overview of some robust statistical methods and how we used these methods in two stages of crystallographic data analysis: peak-finding and bad pixel detection. We introduce a software library called *RGFlib* in which these robust statistical methods are implemented. The software library is adaptable to the outlier-detection tasks in SX data analysis and can be used by relevant data-analysis software suits.

We discussed how robust statistical methods and *RGFlib* can be used in the task of Bragg peak-finding for robust modelling of the background of diffraction patterns and in order to segment pixel intensities into background (true data points) and Bragg peaks (outliers). The modelling of the background needs to be accurate in the presence of Bragg peaks that are outliers compared with the background model. A performance evaluation and the benefits of using robust methods for peak-finding have been reported in our previous publication (Hadian-Jazi *et al.*, 2021 ▸).

Bad pixel detection is a vital task in the SX data-analysis pipeline, since bad pixels can have intensities similar to Bragg peaks and therefore peak-finding methods detect bad pixels as Bragg peaks. In data sets where there are no Bragg peaks, such as data sets collected when the detector is under a dark or a near-flat bright field, all pixels should behave similarly. *RMM* uses robust statistical methods to compare the statistical features of pixels with each other to detect pixels that are behaving abnormally. The *RMM* method has been evaluated with multiple data sets collected using different detectors and has shown high performance in the detection of bad pixels, leading to effective data reduction.

In summary, robust statistical methods are accurate and reliable and they can improve the SX data-analysis pipeline (Hadian-Jazi *et al.*, 2017 ▸, 2021 ▸; Sadri *et al.*, 2022 ▸). 

## Footnotes

1 This package is most efficient for Linux-based OS where forking processes are supported.
